# Molecular characterization of the CXCR4 / CXCR7 axis in germ cell tumors and its targetability using nanobody-drug-conjugates

**DOI:** 10.1186/s40164-023-00460-9

**Published:** 2023-11-23

**Authors:** Gamal A. Wakileh, Philipp Bierholz, Mara Kotthoff, Margaretha A. Skowron, Felix Bremmer, Alexa Stephan, Stephanie M. Anbuhl, Raimond Heukers, Martine J. Smit, Philipp Ströbel, Daniel Nettersheim

**Affiliations:** 1 Department of Urology, Urological Research Laboratory, Translational UroOncology, Medical Faculty and University Hospital Düsseldorf, Heinrich Heine University Düsseldorf, Moorenstraße 5, 40225 Düsseldorf, Germany; 2 Department of Urology, University Hospital Ulm, Ulm, Germany; 3 Institute of Pathology, University Medical Center Göttingen, Göttingen, Germany; 4 Amsterdam Institute for Molecular and Life Sciences, Division of Medicinal Chemistry, Faculty of Sciences, Vrije Universiteit, Amsterdam, Netherlands; 5 QVQ Holding BV, Utrecht, the Netherlands; 6 Center for Integrated Oncology Aachen Bonn Cologne Düsseldorf (CIO ABCD), Düsseldorf, Germany

**Keywords:** Germ cell tumors, Testis cancer, Therapy, Resistance, Nanobody-drug-conjugate, CXCR4, CXCR7, CXCL12

## Abstract

Being stimulated by the chemokine CXCL12, the CXCR4 / CXCR7 cascade is involved in tumor proliferation, migration, and metastasis. The interaction between CXCL12, secreted by cells from the microenvironment, and its receptors is complex and has been ascribed to promote chemotherapy resistance. However, the role of this signaling axis and its targetability in germ cell tumors (GCT) is not fully understood. Thus, this study investigated the therapeutic efficacy of a nanobody-drug-conjugate targeting CXCR4 (CXCR4-NDC) and functionally characterized this signaling pathway in GCT using small molecule inhibitors and nanobodies. As shown by diminished cell viability, enhanced apoptosis induction, and detection of mitotic catastrophes, we confirmed the cytotoxic efficacy of the CXCR4-NDC in CXCR4^+^-GCT cells (i.e. seminoma and yolk-sac tumor), while non-malignant CXCR4^−^-fibroblasts, remained largely unaffected. Stimulation of CXCR4^+^ / CXCR7^+^-GCT cells with CXCL12 resulted in an enhanced proliferative and migratory capacity, while this effect could be reverted using CXCR4 inhibitors or a CXCR7-nanobody. Molecularly, the CXCR4 / CXCR7-signaling cascade could be activated independently of MAPK (ERK1 / 2)-phosphorylation. Although, in CXCR4^−^ / CXCR7^−^-embryonal carcinoma cells, *CXCR7*-expression was re-induced upon inhibition of ERK1 / 2-signaling. This study identified a nanobody-drug-conjugate targeting CXCR4 as a putative therapeutic option for GCT, i.e. seminoma and yolk-sac tumors. Furthermore, this study shed light on the functional role of the CXCR4 / CXCR7 / CXCL12-signaling cascade in GCT, demonstrating an important influence on proliferation and migration.

**Supplementary Information:**

The online version contains supplementary material available at 10.1186/s40164-023-00460-9.

## To the editor

GCT account for 1–2% of all neoplasms occurring in the male population, but represent the most common cancer type in young men [1]. The CXCR4 / CXCR7 / CXCL12-cascade has been postulated to play a major role during metastasis in GCT. In this study, we therapeutically targeted this axis in GCT. Further, we characterized the molecular role of this cascade.

**Fig. 1:**
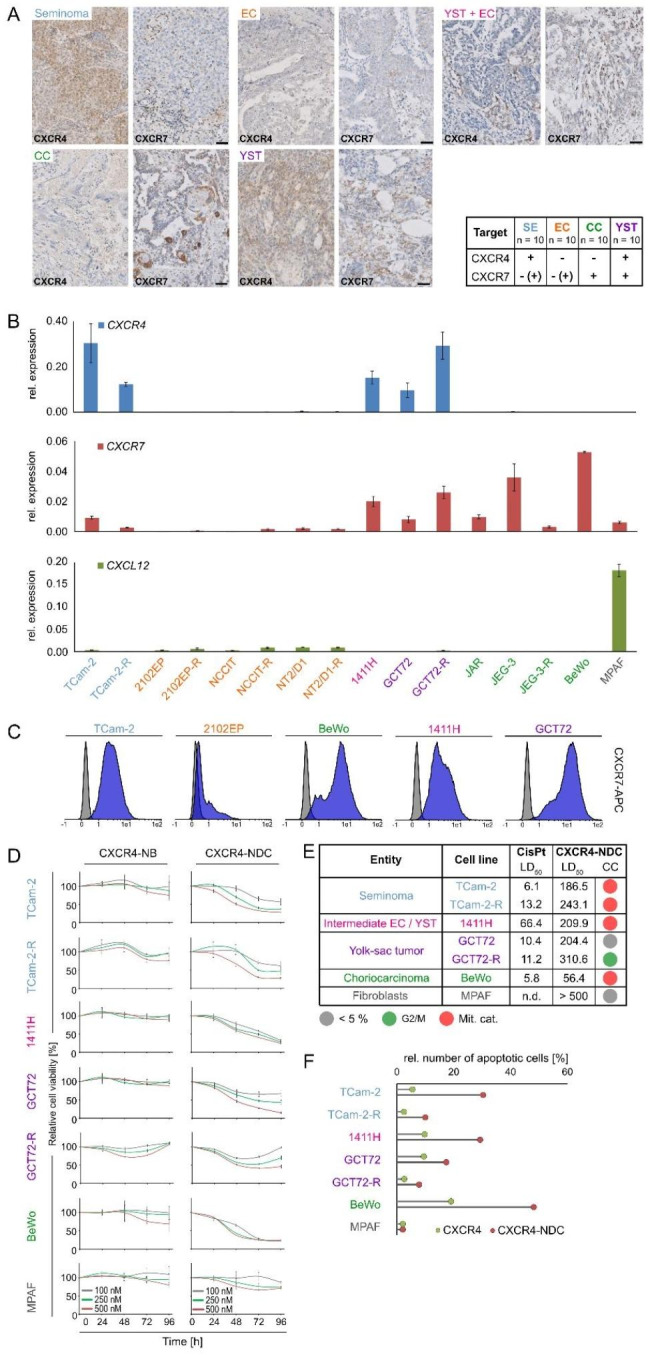
(**A**) Immunohistochemical evaluation of CXCR4 / CXCR7 in SEM, CC, YST and YST + EC mixed GCT. (**B**) Relative expression of *CXCR4*, *CXCR7*, and *CXCL12* in GCT cell lines and fibroblasts (MPAF). *ACTB* and *GAPDH* were used as housekeeping genes. (**C**) Flow cytometry data of CXCR7-APC stained GCT cell lines (blue) compared with unstained controls (grey). (**D**) XTT cell viability assays of GCT cell lines and fibroblasts (MPAF) treated with CXCR4-NDC or CXCR4-NB alone for 24–96 h. (**E**) LD_50_ values measured by XTT cell viability assays 72 h after treatment with cisplatin (CisPt, µM) or CXCR4-NDC (nM) and color-coded changes in cell cycle distribution (mitotic catastrophe = red; changes < 5% = grey) upon treatment with CXCR4-NDC (LD_50_ concentrations) for 72 h as compared to the CXCR4-NB alone. (**F**) Lollipop graph summarizing the relative number of apoptotic cells in CXCR4^+^ GCT cells and CXCR4^−^ MPAF upon treatment with either CXCR4-NDC or CXCR4-NB alone (LD_50_ concentrations) for 72 h

According to ‘The Cancer Genome Atlas’ (TCGA), mutations in *CXCR4* / *CXCR7* were not found in GCT (Fig. S1A). On protein level, seminoma (SEM) tissues presented as CXCR4^+^/ CXCR7^−^, embryonal carcinoma (EC) as CXCR4^−^ / CXCR7^−^, choriocarcinoma (CC) as CXCR4^− ^/ CXCR7^+^, and yolk-sac tumors (YST) as CXCR4^+^ / CXCR7^+^ (Fig. 1A). In mixed GCT (YST + EC), only YST cells were CXCR4^+^ / CXCR7^+^. In GCT cell lines including cisplatin-resistant subclones (-R), similar observations were found on mRNA level (SEM: TCam-2^CXCR4+/CXCR7+^; EC: 2102EP / NT2/D1 / NCCIT^CXCR4−/CXCR7−^; CC: JAR / JEG-3 / BeWo^CXCR4−/CXCR7+^; YST: GCT72^CXCR4+/CXCR7+^; intermediate EC / YST: 1411H^CXCR4+/CXCR7+^) (Fig. 1B; Fig. S1B). *CXCR7* expression profiles could be confirmed on protein level (Fig. 1C). Previously, on protein level we already demonstrated that SEM and YST cells present as CXCR4^+^, while EC and CC cells were CXCR4^−^ [2]. Fibroblasts (MPAF) showed negligible *CXCR4* / *CXCR7* levels, while expressing and secreting CXCL12 (Fig. 1B; Fig. S1C).

We therapeutically targeted CXCR4 by a nanobody coupled to the spindle-toxin monomethyl-auristatin-E (nanobody-drug-conjugate; NDC) [3]. Treatment of SEM and YST cells with the CXCR4-NDC decreased cell viability compared to the uncoupled CXCR4-nanobody (CXCR4-NB) (Fig. 1D, E). The LD_50_ concentrations for the CXCR4-NDC were between 56.4 and 310.6 nM for GCT cells (including cisplatin-resistant sub clones (-R)) and > 500 nM for fibroblasts, thereby opening a therapeutic window (Fig. 1E). A CXCR4-NDC treatment mainly resulted in mitotic catastrophes (TCam-2 / -R, 1411H, BeWo) and induction of apoptosis in CXCR4^+^-cells (Fig. 1E, F; Fig. S1D). In fibroblasts, the cell cycle and apoptosis remained unaffected (Fig. 1E, F; Fig. S1D).

**Fig. 2:**
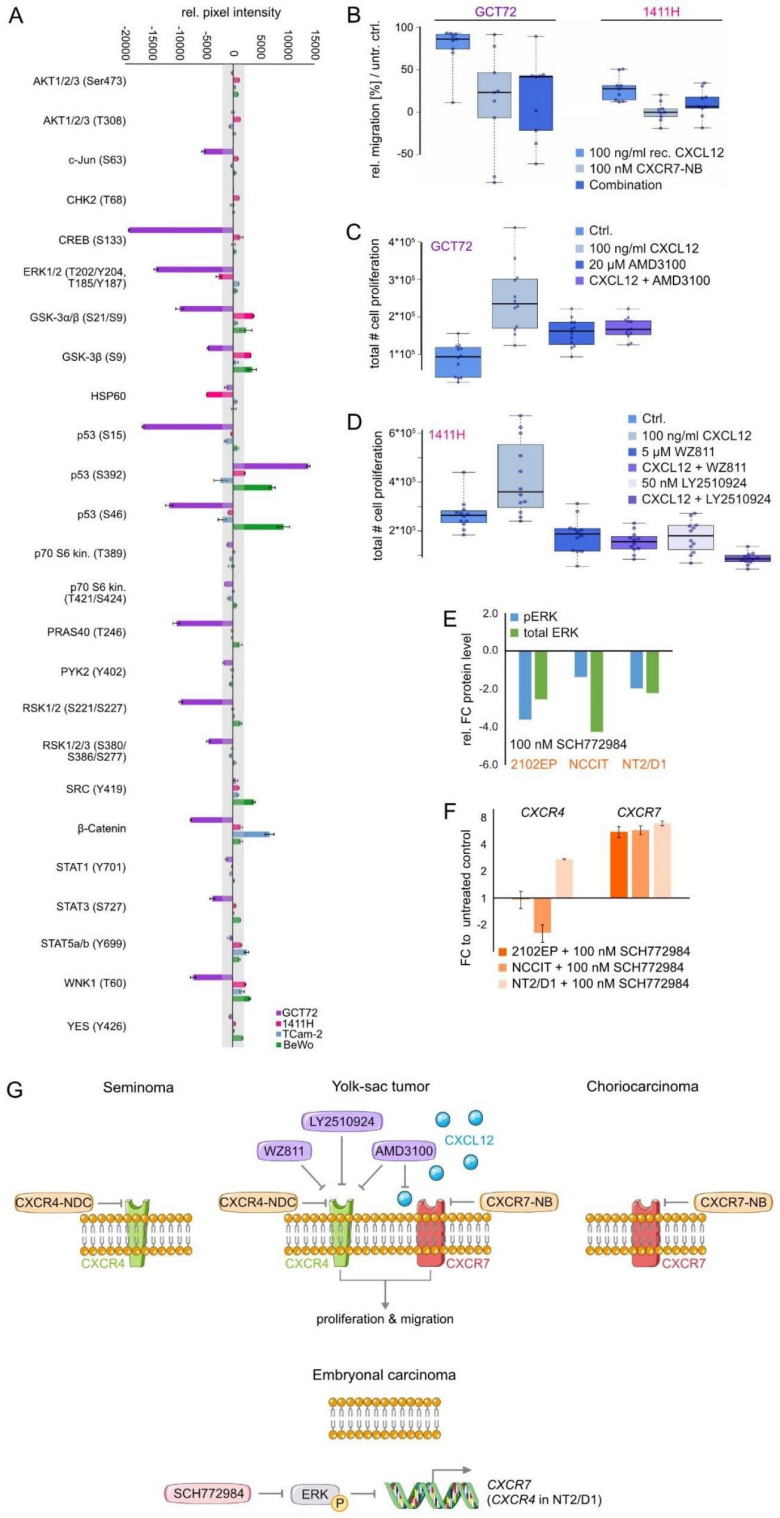
(**A**) Densitometric evaluation of relative pixel intensities (normalized to untreated controls) of the indicated phosphorylation sites in cell lysates from GCT72, 1411H, TCam-2 and BeWo cells treated with recombinant CXCL12 (250 ng / ml) for 24 h, as measured by a human phospho-kinase array. (**B**) Relative migration of GCT72 and 1411H cells treated with either 100 ng / ml recombinant CXCL12, 100 nM CXCR7-NB (VUN702), or the combination of both, in comparison with the untreated control. (**C**) Box plot summarizing the number of proliferative GCT72 cells treated with 100 ng / ml recombinant CXCL12, 20 µM CXCR4-inhibitor AMD3100, or the combination of both for 24 h in comparison to the untreated control. (**D**) Box plot summarizing the number of proliferative 1411H cells treated with 100 ng / ml recombinant CXCL12, CXCR4-inhibitors WZ811 (5 µM) / LY2510924 (50 nM), or the combination of both for 32 h in comparison to the untreated control. (**E**) Densitometric evaluation of western blot data of phospho- and total-ERK in EC cells (2102EP, NCCIT, NT2/D1) treated daily with 100 nM ERK inhibitor SCH772984 for 96 h. (**F**) Relative mRNA expression of *CXCR4* and *CXCR7* in EC cell lines (2102EP, NCCIT, NT2/D1) treated daily with 100 nM SCH772984 for 96 h as compared to untreated controls. *ACTB* and *GAPDH* were used as housekeeping genes. (**G**) Model summarizing key findings related to the CXCR4 / CXCR7 / CXCL12 axis. SEM present as CXCR4^+ ^/ CXCR7^−^, YST as CXCR4^+ ^/ CXCR7^+^, CC as CXCR4^− ^/ CXCR7^+^ and EC as CXCR4^− ^/ CXCR7^−^. SEM (also with occult YST subpopulations), YST and CC are targetable by CXCR4- and / or CXCR7-NDC, respectively. CXCL12 stimulated CXCR4 / CXCR7 enhanced proliferation and migration in YST cells. In EC cell lines, inhibition of MAPK (ERK1 / 2) signaling allows for re-induction of *CXCR7* expression (and partly *CXCR4*)

Next, we deciphered the molecular role of this signaling axis on mRNA and phospho-proteome level. Treatment with recombinant CXCL12 (100 ng / ml, 8 h) resulted in enhanced levels of *FOS* and *MMP3* in GCT72^CXCR4+/CXCR7+^ and BeWo^CXCR7+^ cells, while BeWo cells further showed increased expression of *CD44*, *IL6*, *ITGA4A*, and *MMP1* (Fig. S1E). Further, phospho-kinase-arrays were performed in CXCR4^+^ / CXCR7^+^ cells 24 h after treatment with recombinant CXCL12 (250 ng / ml) (Fig. 2A; Fig. S1F). In TCam-2^CXCR4+^ we found increased phosphorylation (p-) of β-Catenin and STAT5a / b (Y699), in 1411H^CXCR4+/CXCR7+^ elevated p-GSK-3α / β (S21 / S9), p-p53 (S392) and p-WNK1 (T60), and in BeWo^CXCR7+^ enhanced levels of p-GSK-3α / β (S21 / S9), p-p53 (S392 / S46), p-SRC (Y419) and p-WNK1 (T60). In GCT72^CXCR4+/CXCR7+^ cells, phosphorylation of thirteen signaling-molecules, including ERK1 / 2 (T202 / Y204, T185 / Y187), GSK-3α / β (S21 / S9), β-Catenin, and p53 (S15 / S46) was reduced, while p-p53 (S392) was strongly enhanced. Phosphorylation of p53 at S15 has been described to increase stability of p53 by blocking MDM2 binding [4, 5]. Similarly, phosphorylation of S392 is also known to stabilize p53, influence its mitochondrial translocation and a transcription-independent apoptotic function. At a later time-point of DNA damage, S46 phosphorylation initiates the p53-mediated apoptosis through the induction of pro-apoptotic genes, such as *p53AIP1* [4, 5]. Hence, in CXCL12-stimulated BeWo cells, p53 phosphorylation at S15 and S46 might indicate an enhanced and stabilized pro-apoptotic p53 signaling cascade. Vice versa, CXCL12-stimulated GCT72 cells revealed a contrary phosphorylation pattern with diminished S15 and S46 phosphorylation of p53. Generally, the basal phosphorylation of all three evaluated p-p53 sites, particularly S46 and S392, is significantly higher in BeWo cells as compared to GCT72 (Fig. S1F), thereby indicating a more profound apoptosis signaling in BeWo cells upon treatment with the CXCR4-NDC (Fig. 1E, F). Thus, reduced p-p53-S15 / S46 after CXCL12-stimulation might diminish the activity of the apoptotic cascade in YST cells, thereby contributing to the high resistance of YST cells to cisplatin.

Furthermore, the migratory and proliferative capacity of GCT72^CXCR4+/CXCR7+^ and 1411H^CXCR4+/CXCR7+^ cells was enhanced upon CXCL12-stimulation; this effect could be blocked upon application of a CXCR7-blocking nanobody (CXCR7-NB) or CXCR4-inhibitors (AMD3100, WZ811, LY2510924) (Fig. 2B-D, G). The CXCR4 / CXCR7-function after CXCL12 stimulation might also be mediated by receptor-heterodimerization [6, 7]. In YST ^CXCR4+/CXCR7+^ cells, we observed that blocking CXCR4 / CXCR7, abolished the effects on proliferation and migration, suggesting that both receptors act in concert to mediate their molecular functions.

We noted a discrepancy between a described CXCL12-dependent MAPK activation and our observed decreased MAPK signaling in CXCL12-stimulated cells [8–10]. Thus, we questioned, if ERK1 / 2 inhibition would influence *CXCR4* / *CXCR7* expression in EC^CXCR4−/CXCR7−^ cells. Indeed, the ERK1 / 2 inhibitor SCH772984 decreased levels of total- and p-ERK1 / 2 (T202 / Y204-T185 / Y187), while increasing *CXCR7* expression (and *CXCR4* in NT2/D1) (Fig. 2E, F; Fig. S1F). Thus, in EC cells, *CXCR7* expression seems to be suppressed by MAPK-signaling, while in YST, diminished MAPK signaling after CXCL12 stimulation seems to allow for *CXCR7* expression (Fig. 2G).

In summary, we highlighted the CXCR4-NDC as a therapeutic option for CXCR4^+^ SEM and YST (Fig. 2G). In YST and CC, also CXCR7 is a putative target, since an uncoupled CXCR7-NB was able to block the CXCR7-mediated molecular effects and proved to be functional. Thus, using a combination of CXCR4- and CXCR7-NDC could be advantageous for the treatment of mixed GCT consisting of SEM, YST and CC. The ability to target YST is of paramount interest, since YST represent the most aggressive GCT entity responsible for a majority of GCT-related death. Under chemotherapy, development of YST represents an escape mechanism, resulting in therapy-resistant relapses. Additionally, about 5% of metastatic SEM present as aggressive relapses accompanied by elevated AFP level, pointing at a YST-subpopulation [11]. In this setting, using a CXCR4-NDC would be beneficial to target both, SEM and the occult YST cells, by rendering the therapy more efficient and reducing the risk of a relapse. Due to tumor heterogeneity, the clinical need to explore tumor subtype-specific targets remains. As such, we identified CD24 to be a specific target for the treatment of EC using NK-CD24-CAR cells [12]. Moreover, we identified the tight-junction molecule CLDN6 as a putative target for the treatment of SEM, EC, CC, and partly YST by using a CLDN6-antibody-drug-conjugate [13]. As such, depending on the tumor subtype, a combined (immuno)therapeutic option should be considered for the treatment of heterogeneous tumors.

### Electronic supplementary material

Below is the link to the electronic supplementary material.

Supplementary Material 1: Fig. S1: A) Mutational status and mRNA expression profile of *CXCR4*, and *CXCR7* / *ACKR3* in the GCT-TCGA cohort. B) Expression profile (RPKM) of *CXCR4* / *CXCR7* in GCT cells based on previously published RNA sequencing data (GSE189472, GSE190792, GSE190022, GSE168646, and GSE195794). C) CXCL12-ELISA of supernatants from fibroblasts (MPAF) and GCT cells NCCIT and JAR. D) Flow cytometry data indicating changes in the cell cycle distribution of GCT cell lines and MPAF treated with the CXCR4-NBC (blue) in comparison to CXCR4-NB controls (grey). E) Expression of *BCL2*, *CD44*, *COL3A1*, *CCND1*, *EGFR*, *FOS*, *IL6*, *ITGA4*, *MMP1/2/3*, *SRC*, *TGFB1/2/3*, *VEGFA* in CXCL12-stimulated (100 ng / ml, 8 h) GCT72, 1411H, TCam-2, and BeWo cells as compared to their respective untreated controls. F) Raw data of a human phospho-kinase array of various cell lysates (GCT72, 1411H, TCam-2, BeWo) treated with recombinant CXCL12 (250 ng / ml) for 24 h. Untreated cells served as controls. G) Western blot analyses of pERK and total ERK of EC cells (2102EP, NCCIT, NT2/D1) treated daily with 100 nM ERK inhibitor SCH772984 for 96 h

Supplementary Material 2: Supplemental ‘Material & Methods’

Supplementary Material 3: Table S1: A) cell lines, B) drugs, C) antibodies, and D) oligonucleotides used in this study

## Data Availability

All data generated or analyzed during this study are included in this published article and its supplementary information files.

## List of abbreviations

ABTS: 2,2'-azino-bis(3-ethylbenzothiazoline-6-sulfonic acid
ADC: Antibody-drug-conjugate
CC: Choriocarcinoma
EC: Embryonal carcinoma
ELISA: Enzyme-linked immunosorbent assay
FFPE: Formalin-fixed, paraffin-embedded
GCT: Germ cell tumor
h: hour
LD_50_: Lethal dose, 50%
min: minutes
MMAE: Monomethyl auristatin E
NB: Nanobody
NDC: Nanobody
NB: Nanobody-drug conjugate
nonSEM: Non-seminoma
p-: Phosphorylation
PCA: Principal component analysis
RPKM: Reads per kilobase million
SEM: Seminoma
TCGA: The Cancer Genome Atlas
YST: Yolk-sac tumor
XTT: 2,3-bis-(2-methoxy-4-nitro-5-sulfophenyl)-2 H-tetrazolium-5-carboxanilide

## References

[1] Znaor A, Lortet-Tieulent J, Jemal A, Bray F. International variations and trends in testicular cancer incidence and mortality. Eur Urol. 2014;65(6):1095–106. 10.1016/j.eururo.2013.11.004 24268506

[2] Skowron MA, Becker TK, Kurz L, Jostes S, Bremmer F, Fronhoffs F (2021). The signal transducer CD24 suppresses the germ cell program and promotes an ectodermal rather than mesodermal cell fate in embryonal carcinomas. Mol Oncol.

[3] Johansson MP, Maaheimo H, Ekholm FS (2017). New insight on the structural features of the cytotoxic auristatins MMAE and MMAF revealed by combined NMR spectroscopy and quantum chemical modelling. Sci Rep.

[4] Liu Y, Tavana O, Gu W (2019). p53 modifications: exquisite decorations of the powerful guardian. J Mol Cell Biol.

[5] Dai C, Gu W (2010). p53 post-translational modification: deregulated in tumorigenesis. Trends Mol Med.

[6] Koch C, Engele J (2020). Functions of the CXCL12 receptor ACKR3/CXCR7-What has been Perceived and what has been overlooked. Mol Pharmacol.

[7] Levoye A, Balabanian K, Baleux F, Bachelerie F, Lagane B (2009). CXCR7 heterodimerizes with CXCR4 and regulates CXCL12-mediated G protein signaling. Blood.

[8] Santagata S, Ieranò C, Trotta AM, Capiluongo A, Auletta F, Guardascione G (2021). CXCR4 and CXCR7 signaling pathways: a focus on the Cross-talk between Cancer cells and Tumor Microenvironment. Front Oncol.

[9] Shi Y, Riese DJ, Shen J (2020). The role of the CXCL12/CXCR4/CXCR7 Chemokine Axis in Cancer. Front Pharmacol.

[10] Huynh C, Dingemanse J, Meyer zu Schwabedissen HE, Sidharta PN (2020). Relevance of the CXCR4/CXCR7-CXCL12 axis and its effect in pathophysiological conditions. Pharmacol Res.

[11] Wruck W, Bremmer F, Kotthoff M, Fichtner A, Skowron MA, Schönberger S (2021). The pioneer and differentiation factor FOXA2 is a key driver of yolk-sac tumour formation and a new biomarker for paediatric and adult yolk-sac tumours. J Cell Mol Med.

[12] Söhngen C, Thomas DJ, Skowron MA, Bremmer F, Eckstein M, Stefanski A, et al. CD24 targeting with NK-CAR immunotherapy in testis, prostate, renal and (luminal-type) Bladder cancer and identification of direct CD24 interaction partners. FEBS J. 2023;290(20):4864–876. 10.1111/febs.16880 PMC11129509 37254618

[13] Skowron MA, Kotthoff M, Bremmer F, Ruhnke K, Parmaksiz F, Richter A (2023). Targeting CLDN6 in germ cell tumors by an antibody-drug-conjugate and studying therapy resistance of yolk-sac tumors to identify and screen specific therapeutic options. Mol Med.

