# Supplementary figures and images for: Molecular characterization of the CXCR4 / CXCR7 axis in germ cell tumors and its targetability using nanobody-drug-conjugates
Source: Exp Hematol Oncol. 2023 Nov 23;12:96. doi: 10.1186/s40164-023-00460-9 (PMC10668499; doi:10.1186/s40164-023-00460-9)

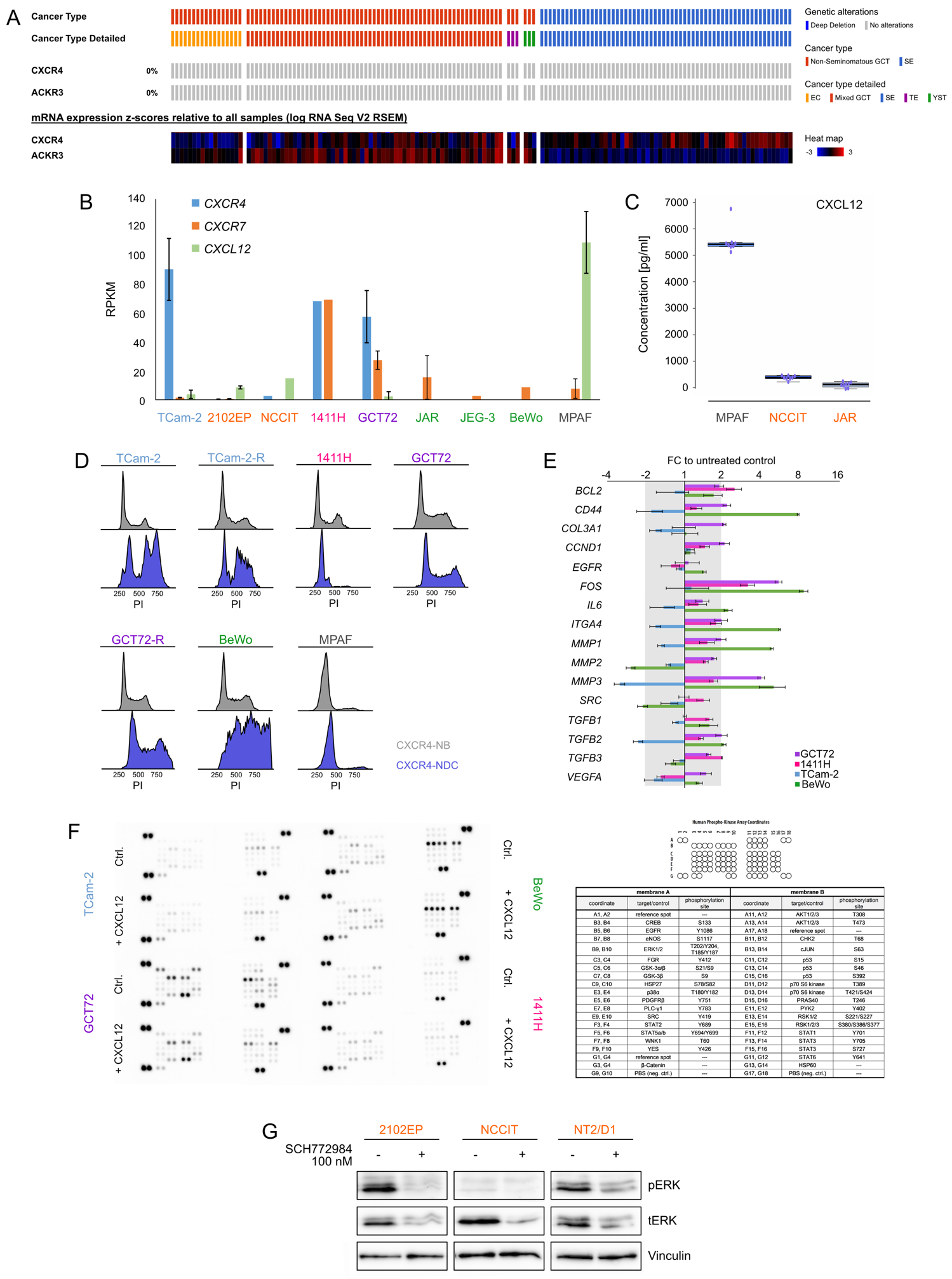

Supplement: Supplementary file 1 — Supplementary Material 1: Fig. S1: A) Mutational status and mRNA expression profile of CXCR4, and CXCR7 / ACKR3 in the GCT-TCGA cohort. B) Expression profile (RPKM) of CXCR4 / CXCR7 in GCT cells based on previously published RNA sequencing data (GSE189472, GSE190792, GSE190022, GSE168646, and GSE195794). C) CXCL12-ELISA of supernatants from fibroblasts (MPAF) and GCT cells NCCIT and JAR. D) Flow cytometry data indicating changes in the cell cycle distribution of GCT cell lines and MPAF treated with the CXCR4-NBC (blue) in comparison to CXCR4-NB controls (grey). E) Expression of BCL2, CD44, COL3A1, CCND1, EGFR, FOS, IL6, ITGA4, MMP1/2/3, SRC, TGFB1/2/3, VEGFA in CXCL12-stimulated (100 ng / ml, 8 h) GCT72, 1411H, TCam-2, and BeWo cells as compared to their respective untreated controls. F) Raw data of a human phospho-kinase array of various cell lysates (GCT72, 1411H, TCam-2, BeWo) treated with recombinant CXCL12 (250 ng / ml) for 24 h. Untreated cells served as controls. G) Western blot analyses of pERK and total ERK of EC cells (2102EP, NCCIT, NT2/D1) treated daily with 100 nM ERK inhibitor SCH772984 for 96 h [file 40164_2023_460_MOESM1_ESM.tiff]
