# Supplementary material for: Molecular characterization of the CXCR4 / CXCR7 axis in germ cell tumors and its targetability using nanobody-drug-conjugates
Source: Exp Hematol Oncol. 2023 Nov 23;12:96. doi: 10.1186/s40164-023-00460-9 (PMC10668499; doi:10.1186/s40164-023-00460-9)
Supplement: Supplementary file 2 — Supplementary Material 2: Supplemental ‘Material & Methods’ [file 40164_2023_460_MOESM2_ESM.docx]

Molecular characterization of the CXCR4 / CXCR7 axis in germ cell tumors and its targetability using nanobody-drug-conjugates

Gamal A. Wakileh^1,2^, Philipp Bierholz^1^, Mara Kotthoff^1^, Margaretha A. Skowron^1^, Felix Bremmer^3^, Alexa Stephan^1^, Stephanie Anbuhl^4, 5^, Raimond Heukers^4, 5^, Martine J. Smit^4^, Philipp Ströbel^3^, Daniel Nettersheim^1+^

^1^ Department of Urology, Urological Research Laboratory, Translational UroOncology, Medical Faculty and University Hospital Düsseldorf, Heinrich Heine University Düsseldorf, Germany

^2^ Department of Urology, University Hospital Ulm, Ulm, Germany

^3^ Institute of Pathology, University Medical Center Göttingen, Göttingen, Germany

^4^ Amsterdam Institute for Molecular and Life Sciences, Division of Medicinal Chemistry, Faculty of Sciences, Vrije Universiteit, Amsterdam, Netherlands

^5^ QVQ Holding BV, Utrecht, the Netherlands.

**Supplemental ‘Material & Methods’**

Cell culture

GCT cell lines, as well as their cisplatin resistant sublines (-R), and fibroblasts (MPAF) were kept under culture conditions as described in Table S1A and were checked for *Mycoplasma* contamination as well as authenticity (short tandem repeats (STR) profiles). For the generation of a cisplatin resistant subclone of the YST cell line GCT72, cells were exposed to cisplatin once per month for a total period of 20 month.

Development of nanobody-drug-conjugates

The generation of CXCR4-NB (VUN401), CXCR4-NDC (VUN401-MMAE) and CXCR7/ACKR3-Flag-His-NB (VUN702) has been described previously (QVQ Holding BV, Utrecht, The Netherlands) ^1–3^. VHHs were produced by *E. coli* strain TG1 or *S. cerevisiae* strain VWK18 and were purified using His / Co IMAC or protein affinity chromatography, respectively.

XTT cell viability assays

To evaluate the influence of the CXCR4-NB and CXCR4-NDC on cell viability, 3 x 10^3^ cells were seeded in quadruplicates into 96-well plates. After 24 h, CXCR4-NB or CXCR4-NDC was applied and relative cell viability was evaluated over a period of 24 - 96 h using XTT (2,3-bis-(2-methoxy-4-nitro-5-sulfophenyl)-2H-tetrazolium-5-carboxanilide) and phenazine methosulfate as described previously ^4–6^. After an incubation of 4 h, the absorbance (450 nm / 655 nm) was measured using the iMark Microplate Absorbance Reader (BioRad, Feldkirchen, Germany).

Transwell migration and proliferation assay

Transwell migration assays were performed as described previously after stimulation of tumor cells with recombinant human 100 ng / ml CXCL12 (Abcam, Cambridge, United Kingdom) in combination with 100 nM CXCR7-NB ^7^. For the evaluation of proliferative capacity, cells were treated with 100 ng / ml CXCL12 (Abcam) in combination with the CXCR4 inhibitors AMD3100, LY2510924, or WZ811. See table S1 B for detailed information on utilized recombinant proteins and inhibitors (Table S1 B).

Phospho-kinase array

For the evaluation of phosphorylated kinases, cells were stimulated with 250 ng / ml CXCL12 (Peprotech via Biozol Diagnostica Vertrieb GmbH, Eiching, Germany) for 24 hours (h), before being harvested. The ‘Proteome Profiler Human Phospho-Kinase Array Kit’ (R&D Systems via Bio-Techne, Wiesbaden, Germany) was carried out according to the manufacturer’s protocol using 400 µg of protein per array. The ChemiDoc Imaging System (BioRad) was used for detection, while the ‘Protein Array Analyzer’-Plugin for ‘Image J’ (https://imagej.nih.gov/ij/) was utilized for the densitometric analysis of the spots ^8,9^.

CXCL12 ELISA

The human ‘CXCL12 Standard ABTS ELISA Development Kit’ (Peprotech) was performed according to the manufacturer’s protocol to evaluate the concentration of CXCL12 in the supernatant of confluent T-75 cell culture flasks.

Flow cytometry

The intracellular antibody-staining protocol was used to determine the number of CXCR7^+^ cells by flow cytometry. Briefly, after fixation of cell suspensions with 4 % formaldehyde for 5 minutes (min), cells were permeabilized with 0.1 % Tween-20 in PBS for 5 min. Staining with 1:100 diluted CXCR7-APC (BioLegend via Biozym, Hessisch Oldendorf, Germany) was performed for 30 min on a rocking platform. See table S1 B for detailed information on the utilized antibodies (Table S1 C). For the evaluation of apoptosis induction and changes in cell cycle distribution, cells were analyzed as described previously ^4–6^. Briefly, 9 x 10^4^ cells were seeded into 6-well plates before being treated with either CXCR4-NB or CXCR4-NDC (LD_50 72 h_) 24 h later. After 72 h, cell suspensions were stained with 2.5 µl Annexin V-FITC (Miltenyi Biotec, Bergisch Gladbach, Germany) and 15 µg propidium iodide (PI, Sigma-Aldrich, Taufkirchen, Germany) in Annexin V-binding buffer (Miltenyi Biotec) for the apoptosis assay. For the cell cycle analysis, cells were washed with PBS (phosphate buffered saline) before fixation in ice cold 70 % ethanol and staining with 2 µg / ml PI and 200 µg / ml RNaseA (Qiagen, Hilden, Germany). For all measurements, at least 5 x 10^4^ cells were counted using the ‘MACSQuant’ flow cytometer, which were later analyzed using the ‘Flowlogic’ software (Miltenyi Biotec).

RNA extraction, cDNA synthesis and quantitative RT-PCR

For determining changes in gene expression via qRT-PCR, mRNA was extracted using the RNeasy kit according to the manufacturers protocol (Qiagen). Subsequently, 1 µg mRNA was *in-vitro* transcribed using Oligo(dT)18 Primer, dNTPs, RiboLock RNAse inhibitor and Maxima H Minus Reverse Transcriptase (200 U / µL) (all Thermo Fisher Scientific, Schwerte, Germany). Using the SYBR-green-based Luna Universal qPCR Master Mix (New England Biolabs, Frankfurt am Main, Germany), 7.34 ng of cDNA per samples (n = 3) was measured using the 384-well C1000 cycler (BioRad). *ACTB* and *GAPDH* were used as housekeeping genes for normalization. See table S1 D for oligonucleotide sequences (Table S1 D).

Western blot analyses

For the evaluation of ERK-inhibition, 5 x 10^4^ EC cells were seeded into 6-well plates before being treated daily with 100 nM SCH770984 (Selleckchem via Biozol) or the solvent control (DMSO, Sigma-Aldrich) for 96 h. As described previously ^4–6^, 20 µg of whole protein lysates were isolated by RIPA buffer (Cell Signaling, Frankfurt am Main, Germany). Protein concentrations were determined using the ‘Pierce BCA Protein Assay-Kit’ according to the manufacturer’s protocol. For SDS-PAGE, the Mini-PROTEAN Electrophoresis system (BioRad) was used, while gels were blotted onto PVDF membranes (Merck KGaA, Darmstadt, Germany) by utilizing the Trans-Blot Turbo system (BioRad). Successful protein transfer was confirmed by staining the PVDF membrane in Ponceau S Solution (Sigma-Aldrich). Upon blocking in 5 % milk in PBS-T (1 x PBS with 1 % Tween-20, both Sigma-Aldrich), antibodies were incubated overnight at 4°C. The next day, membranes were incubated with secondary HRP (horseradish peroxidase)-coupled antibodies for 2 h at room temperature. Afterwards, upon incubation for 5 min in the dark with ‘Pierce ECL Western Blotting Substrate’ (Thermo Fisher Scientific, Schwerte, Germany), chemiluminescent signals were detected by the ChemiDoc Imaging System (BioRad). All utilized antibodies are listed in table S1 (Table S1 C).

Immunohistochemistry

As described previously ^10^, immunohistochemistry has been performed using citrate buffer and incubation of primary antibodies for 30 min at room temperature (RT). Subsequently, ready-to-use HRP-labelled secondary antibodies were incubated for 25 min at RT before being detected using DAB+ (3,3'-Diaminobenzidine) Chromogen system and counterstained with Meyers’s hematoxylin. See table S1 C for antibody details (Table S1 C).

Online analysis tools

The GCT TCGA (‘The Cancer Genome Atlas’) cohort was analyzed using cBioportal (https://www.cbioportal.org/) ^11,12^, FireBrowse (http://firebrowse.org/) ^13^, and the Xena Functional Genomics Explorer (https://xenabrowser.net/) ^14^. ‘The online platforms ‘BoxPlotR’ (http://shiny.chemgrid.org/boxplotr/) was used to generate box plots. Graphical illustrations have been designed using ‘bioicons’ (https://bioicons.com/).

**References**

1. Bobkov, V. *et al.* Nanobody-Fc constructs targeting chemokine receptor CXCR4 potently inhibit signaling and CXCR4-mediated HIV-entry and induce antibody effector functions. *Biochem. Pharmacol.* **158**, 413–424 (2018).

2. Van Hout, A. *et al.* CXCR4-targeting nanobodies differentially inhibit CXCR4 function and HIV entry. *Biochem. Pharmacol.* **158**, 402–412 (2018).

3. Song, S. *et al.* D-dopachrome tautomerase contributes to lung epithelial repair via atypical chemokine receptor 3-dependent Akt signaling. *EBioMedicine* **68**, 103412 (2021).

4. Burmeister, A. *et al.* Establishment and evaluation of a dual HDAC / BET inhibitor as a therapeutic option for germ cell tumors and other urological malignancies. *Mol. Cancer Ther.* **21**, 1674–1688 (2022).

5. Müller, M. R. *et al.* Therapeutical interference with the epigenetic landscape of germ cell tumors: a comparative drug study and new mechanistical insights. *Clin. Epigenetics 2022 141* **14**, 5 (2022).

6. Skowron, M. A. *et al.* The signal transducer CD24 suppresses the germ cell program and promotes an ectodermal rather than mesodermal cell fate in embryonal carcinomas. *Mol. Oncol.* (2021). doi:10.1002/1878-0261.13066

7. Skowron, M. A. *et al.* Profiling the 3D interaction between germ cell tumors and microenvironmental cells at the transcriptome and secretome level. *Mol. Oncol.* 1–21 (2022). doi:10.1002/1878-0261.13282

8. Schneider, C. A., Rasband, W. S. & Eliceiri, K. W. NIH Image to ImageJ: 25 years of image analysis. *Nat. Methods 2012 97* **9**, 671–675 (2012).

9. Carpentier, G. Protein Array Analyzer. *Accessed 03 Feb 2022*

10. Wruck, W. *et al.* The pioneer and differentiation factor FOXA2 is a key driver of yolk-sac tumour formation and a new biomarker for paediatric and adult yolk-sac tumours. *J. Cell. Mol. Med.* (2021). doi:10.1111/jcmm.16222

11. Gao, J. *et al.* Integrative analysis of complex cancer genomics and clinical profiles using the cBioPortal. *Sci. Signal.* (2013). doi:10.1126/scisignal.2004088

12. Cerami, E. *et al.* The cBio Cancer Genomics Portal: An open platform for exploring multidimensional cancer genomics data. *Cancer Discov.* **2**, 401–404 (2012).

13. Deng, M., Brägelmann, J., Kryukov, I., Saraiva-Agostinho, N. & Perner, S. FirebrowseR: an R client to the Broad Institute’s Firehose Pipeline. *Database (Oxford).* **2017**, (2017).

14. Goldman, M., Craft, B., Brooks, A., Zhu, J. & Haussler, D. Visualizing and interpreting cancer genomics data via the Xena platform. *Nat Biotechnol* **38**, 675–678 (2020).
